# Proteomic Substrate Identification for Membrane Proteases in the Brain

**DOI:** 10.3389/fnmol.2016.00096

**Published:** 2016-10-13

**Authors:** Stephan A. Müller, Simone D. Scilabra, Stefan F. Lichtenthaler

**Affiliations:** ^1^German Center for Neurodegenerative Diseases (DZNE)Munich, Germany; ^2^Neuroproteomics, Klinikum rechts der Isar, Technische Universität MünchenMunich, Germany; ^3^Institute for Advanced Study, Technische Universität MunichGarching, Germany; ^4^Munich Cluster for Systems Neurology (SyNergy)Munich, Germany

**Keywords:** proteomics, degradomics, protease, BACE, ADAM10, ADAM17, Alzheimer’s disease

## Abstract

Cell-cell communication in the brain is controlled by multiple mechanisms, including proteolysis. Membrane-bound proteases generate signaling molecules from membrane-bound precursor proteins and control the length and function of cell surface membrane proteins. These proteases belong to different families, including members of the “a disintegrin and metalloprotease” (ADAM), the beta-site amyloid precursor protein cleaving enzymes (BACE), membrane-type matrix metalloproteases (MT-MMP) and rhomboids. Some of these proteases, in particular ADAM10 and BACE1 have been shown to be essential not only for the correct development of the mammalian brain, but also for myelination and maintaining neuronal connections in the adult nervous system. Additionally, these proteases are considered as drug targets for brain diseases, including Alzheimer’s disease (AD), schizophrenia and cancer. Despite their biomedical relevance, the molecular functions of these proteases in the brain have not been explored in much detail, as little was known about their substrates. This has changed with the recent development of novel proteomic methods which allow to identify substrates of membrane-bound proteases from cultured cells, primary neurons and other primary brain cells and even *in vivo* from minute amounts of mouse cerebrospinal fluid (CSF). This review summarizes the recent advances and highlights the strengths of the individual proteomic methods. Finally, using the example of the Alzheimer-related proteases BACE1, ADAM10 and γ-secretase, as well as ADAM17 and signal peptide peptidase like 3 (SPPL3), we illustrate how substrate identification with novel methods is instrumental in elucidating broad physiological functions of these proteases in the brain and other organs.

## Proteolytic Processing in Alzheimer’S Disease

Proteolysis is a biological process playing an essential role in all organisms and tissues, including the brain. For example, proteolysis regulates numerous cell functions, spanning from degradation of faulty proteins to post-translational generation of active signaling molecules, neurite outgrowth and modeling of the extracellular matrix. Therefore, protease activity must be tightly regulated and, conversely, aberrant proteolysis is associated with several pathological conditions ranging from inflammation to cancer and neurodegeneration. A prime example is Alzheimer’s disease (AD), where deregulation of proteolysis leads to neurodegeneration. AD is the most common type of dementia, a syndrome characterized by loss of memory and cognitive decline. AD causes a substantial loss of neurons and synapses in the brain, leading to an overall loss in brain weight. Additional neuropathological hallmarks of the disease are the amyloid-β (Aβ) plaques, consisting of the mostly 42 amino acid long Aβ peptide (Aβ42), and the intraneuronal accumulation of neurofibrillary tangles, consisting of hyperphosphorylated forms of the microtubule-associated protein tau (Huang and Mucke, 2012). According to the widely accepted amyloid cascade hypothesis (Selkoe and Hardy, 2016), Aβ forms neurotoxic oligomers, which initiate an inflammatory response involving the activation of microglia and astrocytes. Subsequently tau becomes aberrantly phosphorylated and aggregates in neurofibrillary tangles, leading to synaptic loss, neuronal death, and ultimately dementia (Selkoe and Hardy, 2016).

**Figure 1:**
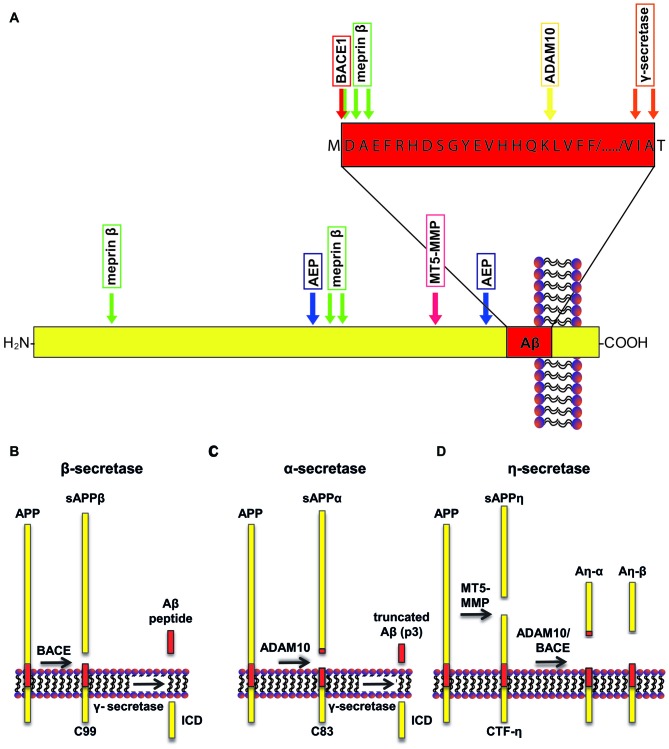
**Schematic representation of amyloid precursor protein (APP) processing. (A)** A number of proteases can cleave APP at specific sites, including a disintegrin and metalloproteinase 10 (ADAM10; yellow arrow), beta-site APP cleaving enzyme 1 (BACE1; red arrow), γ-secretase (orange arrows), asparagine endopeptidase (AEP; blue arrows), membrane-type matrix metalloproteases (MT5-MMP; fuchsia arrow) and meprin β (green arrows). **(B)** APP can undergo amyloidogenic processing when cleaved by BACE1. Cleavage of APP by BACE1 results in generation of sAPPβ. Subsequent cleavage of the remaining transmembrane domain by γ-secretase releases amyloid-β (Aβ). **(C)** Conversely, cleavage of APP by ADAM10 favors the non-amyloidogenic pathway, releasing sAPPα. Subsequent γ-secretase cleavage releases a non-toxic truncated form of the Aβ peptide, called p3. **(D)** In addition, APP can be cleaved by MT5-MMP, which results in the release of sAPPη. Consecutively, C-terminal fragment (CTF)-η can be cleaved by ADAM10 or BACE1 that release Aη-α and Aη-β, respectively. The recently identified δ-secretase cleaves APP a few amino acids N-terminally to the BACE1 cleavage site (not shown in the figure).

Aβ derives from the transmembrane protein amyloid precursor protein (APP; Dislich and Lichtenthaler, 2012; Figure 1A) through sequential cleavage by two proteases, the β- and γ-secretase (Haass and Selkoe, 2007). The β-secretase was identified in 1999 by five independent research groups, and is referred to as β-site APP cleaving enzyme 1 (BACE1; Hussain et al., 1999; Sinha et al., 1999; Vassar et al., 1999; Yan et al., 1999; Lin et al., 2000). BACE1 cleavage releases a soluble extracellular fragment of APP (sAPPβ) and generates a carboxy (C)-terminal membrane-tethered fragment known as C99 (Figure 1). C99 undergoes a subsequent intramembrane cleavage by γ-secretase, a multi-subunit protease complex comprising four transmembrane proteins: presenilin, nicastrin, Pen2 and Aph1 (De Strooper et al., 2010). The γ-secretase cleavage of C99 generates Aβ and releases intracellularly the APP intracellular domain (AICD). APP can undergo an alternative cleavage, mediated by a disintegrin and metalloproteinase 10 (ADAM10; Lammich et al., 1999; Kuhn et al., 2010), also known as α-secretase, that releases its soluble ectodomain (sAPPα) and generates a membrane-tethered fragment, C83 (Figure 1B). Importantly, the subsequent cleavage by γ-secretase releases a truncated form of Aβ, which is non-toxic. Three other proteases emerged to be involved in the processing of APP. Asparagine endopeptidase (AEP), known as the δ-secretase, is a cysteine proteinase that mediates APP processing in an age-dependent manner and is linked to AD pathogenesis (Zhang et al., 2015). Furthermore, the membrane-tethered metalloproteinase (MT5-MMP) cleaves APP at amino acids 504–505, initiating a proteolytic processing that leads to the generation of APP fragments (Aη-α), which lower neuronal activity (Ahmad et al., 2006; Willem et al., 2015; Figure 1C). Loss of MT5-MMP ameliorates pathology and behavioral deficits in a mouse model of AD (Baranger et al., 2016). A member of the meprin family of metalloproteases, meprin β, was also shown to cleave APP, with the cleavage site being identical to that of the β-secretase or in close proximity to it. This shedding event is followed by the γ-secretase cleavage and leads to the generation of Aβ or truncated variants of Aβ (i.e., Aβ^ 2–40^; Bien et al., 2012). Additionally, meprin β can process APP at the N-terminus, releasing two N-terminal fragments of APP of 11 and 22 kDa, namely APP11 and APP22 (Jefferson et al., 2011).

## Regulated Intramembrane Proteolysis

**Figure 2:**
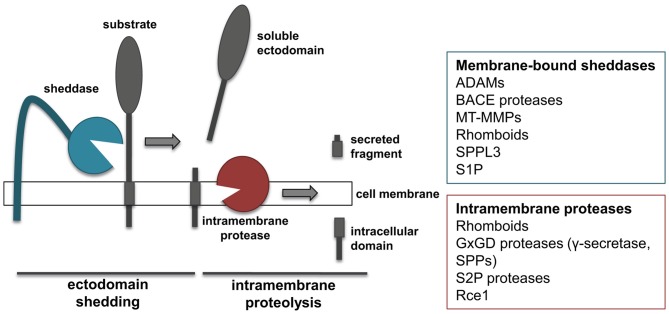
**“Shedding” and “RIPping”.** Schematic representation of ectodomain shedding and regulated intramembrane proteolysis (RIP), including a list of protease families known to function as sheddases or intramembrane proteases.

The proteolytic processing of APP is a prime example for a proteolytic process referred to as regulated intramembrane proteolysis (RIP; Figure 2). RIP frequently comprises two proteolytic cleavages, namely shedding and intramembrane proteolysis. Shedding is mediated by membrane-tethered proteases, referred to as “sheddases”, which cleave their transmembrane substrates, thereby releasing their soluble ectodomains into the extracellular milieu (Figure 2). Most sheddases cleave their substrates at peptide bonds outside of the membrane, but at a short distance from the lumenal or extracellular membrane surface. Shedding can be followed by a second cleavage within the substrates’ transmembrane domain. This cleavage results in release of the intracellular domain (ICD) into the cytosol and the extracellular secretion of the small remaining peptide. As it occurs for APP, α- and β-secretase function as sheddases, and their activity can be coupled with the action of γ-secretase to perform RIPping of the remaining membrane-tethered protein fragment.

Shedding and intramembrane proteolysis initiate a sequence of extracellular and intracellular events that control a broad range of physiological processes in the brain, including cell-cell communication, cell differentiation and development (Murphy et al., 2008; Lichtenthaler et al., 2011; Weber and Saftig, 2012). For instance, the tumor necrosis factor-α (TNF), a proinflammatory cytokine, is generated as a transmembrane protein that needs to be shed by ADAM17 from the cell surface in order to trigger immune responses (Black et al., 1997). Interestingly, the remaining membrane-bound fragment can be further cleaved by SPPL2a or SPPL2b within the membrane, releasing the TNF ICD which acts as an additional signaling molecule (Friedmann et al., 2006). Similarly to TNF, several growth factors, including EGF-like growth factors and neuregulins, are inactive when bound to the membrane and get activated by proteolytic shedding (Blobel, 2005).

Sheddases do not only modulate the availability of ligands, but also regulate the activity of signaling receptors. Notch is a clear example of cell surface receptor that requires RIPping to initiate its signaling pathway and control cell-differentiation (Hartmann et al., 2002). For other substrates, RIP is a mechanism to terminate a protein’s function. For example, shedding shuts down the signaling function of TNF receptors (D’Alessio et al., 2012; Deng et al., 2015) or the adhesive functions of cell adhesion proteins (Solanas et al., 2011).

## Sheddases and Intramembrane Proteases

Members of several different families of proteases have been shown to function as sheddases, including several ADAMs, BACE proteases, membrane-type metalloproteinases (MT-MMPs) and rhomboids (Figure 2; Blobel, 2005; Vassar et al., 2014; Itoh, 2015). In addition, signal peptide peptidase like 3 (SPPL3) from the SPP family and site-1 protease (S1P) can also act as sheddases (Lenz et al., 2001; Voss et al., 2012). ADAM and BACE proteases cleave substrates in their extracellular domain, at a short distance from the membrane, and need the sequential cleavage of an intramembrane proteinase in order to perform RIPping. Conversely, rhomboids and SPPL3 are intramembrane proteases that cleave their substrates within or close to the transmembrane domain. As a consequence of such cleavage, regardless whether it occurs extracellularly or within the transmembrane domain, the ectodomain of substrates is released into the extracellular milieu. This is of note, as the secreted form of transmembrane proteins can acquire functions different from that of the membrane-bound counterpart. MT-MMPs can act as sheddases. However, compared to the related family of ADAMs, MT-MMPs can cleave their substrates more distantly from the cell surface and on different sites, thereby releasing truncated forms of protein ectodomains or lower molecular weight fragments (Selvais et al., 2011; Fu et al., 2013; Willem et al., 2015).

## Function of Proteases is Determined by Substrates

Proteases have been well characterized in pathophysiology of disease as key players in the development of several pathological conditions, including neurodegenerative diseases. Thus, protease inhibition has been widely targeted for drug development. Unfortunately, in the vast majority of cases, therapies based on protease inhibition have failed in clinical trials. Indeed, there are critical limitations to the development of therapies targeting proteases. First, distinct members of a protease family share structural features, thus drug-based inhibition of a specific protease can affect the activity of homologs. For example, BACE1 inhibitors have been developed to reduce Aβ production in the brain and are tested for treatment and prevention of AD. However, they also block the homologous protease BACE2, which has critical functions in pigmentation (Rochin et al., 2013; Neumann et al., 2015). In fact, mice treated with such inhibitors get a gray fur color and patients treated with these drugs need to go for regular dermatology testing (Yan, 2016). More importantly, proteases often do not target a specific substrate, but they can cleave an array of diverse proteins. As a consequence, their inhibition can deregulate a number of cellular processes, and inhibition-based therapies can lead to mechanism-based side effects that often are more pronounced than amelioration of the pathology itself. For instance, due to its central contribution to the pathogenesis of AD, γ-secretase has been extensively targeted for drug development. A number of γ-secretase inhibitors have been generated and tested for their ability to reduce Aβ production *in vitro* and *in vivo*. One of them, called Semagacestat, was terminated in clinical trial Phase III, as it was associated with worsening of patient cognition and with higher incidence of skin cancer (De Strooper, 2014). These mechanism-based side effects were linked to the chronic inhibition of Notch cleavage by γ-secretase.

A deep understanding of protease functions and their roles in cell biology is necessary for developing effective therapeutic strategies. As the biological function of proteases depends on their substrate spectrum, the identification of the substrate repertoire is essential to understand the function of a specific protease and to predict potential side-effects of their therapeutic inhibition. In the last years, a number of proteomics-based methods have been developed in order to identify the substrate repertoire of specific proteases. In this review, we summarize the most commonly used and other suitable methods and give examples of their applications with a focus on sheddases and intramembrane proteases, in particular on BACE1, ADAM10 and γ-secretase in AD.

## Methods for Mass Spectrometry Based Substrate Identification of Membrane Proteases in the Brain

Mass spectrometry (MS) based proteomics offers powerful methods to identify membrane protease substrate candidates *in vitro* and *in vivo*. Especially, non-targeted quantification of protein cleavage products in the secretome of brain-derived primary cells or cell lines, as well as cerebrospinal fluid (CSF) are suitable for protease substrate identification. In this context, the secretome comprises all proteins released by cells into body fluids or into the conditioned medium of cultured cells. For sheddases such as BACE1 and ADAM10, the ectodomain of their substrates is released into the extracellular space. Therefore, usually a loss of function condition, such as protease KO, knockdown (KD), or inhibition, is quantitatively compared with related control conditions to identify substrates. At loss of function conditions, substrate cleavage is fully or partly prevented which leads to a reduced abundance of the related cleavage products in the secretome.

Additionally, some substrates accumulate in the cell membrane when the target protease does not cleave them. Therefore, membrane protease substrate candidates might also be identified by quantitative proteomics due to an increased abundance within the cell membrane. Alternatively, also gain of function conditions such as overexpression of the target protease can be used which leads to increased cleavage activity and subsequently to increased abundance of substrate cleavage products in the secretome.

**Figure 3:**
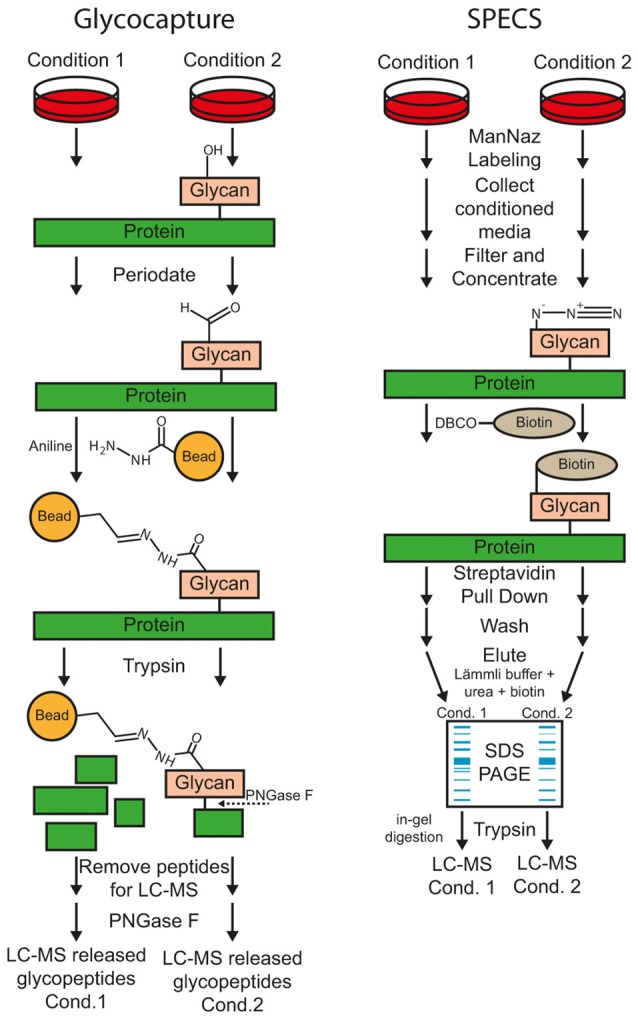
**Workflow of the glyco-capturing and secretome protein enrichment with click sugars (SPECS) method for protease substrate identification**.

**Figure 4:**
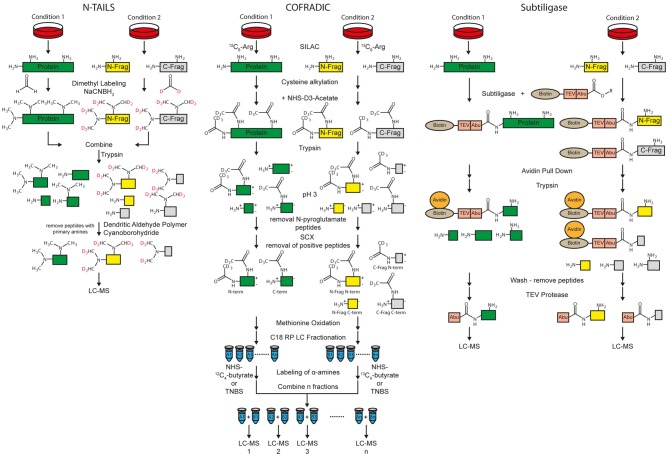
**Workflow of the N-terminal amine-based isotope labeling of substrates (N-TAILS), combined fractional diagonal chromatography (COFRADIC) and subtiligase method for protease substrate identification and cleavage site determination.** Labeling of amines in N-TAILS and COFRADIC can also be performed by isobaric tagging such as iTRAQ or TMT for multiplexing.

Here, we will provide a short overview of the main methods for MS-based protease substrate identification with a focus on methods for sheddase substrate identification. In the first section, methods are described that are used to identify substrates in the secretome or on the cell surface (Figure 3). In the second section, methods are described that also allow protease cleavage site determination (Figure 4). Usually, bottom-up proteomics is used for this purpose. Briefly, in all protocols, secreted or membrane proteins are digested with a protease, usually trypsin, to create proteolytic peptides. In most cases those peptides are separated by C18 reversed phase liquid chromatography (LC) prior to MS analysis. The MS raw data is searched against a protein database to identify proteotypic peptides. Relative peptide and protein quantification can be done by different methods. According to the different protocols for protease substrate identification, label-free and label-based quantification methods are used. A detailed explanation of different quantification methods can be found in several review articles (Bantscheff et al., 2007; Schulze and Usadel, 2010; Bakalarski and Kirkpatrick, 2016).

### Methods for Protease Substrate Identification

#### Glyco-capture

Most substrates of sheddases are single-pass transmembrane or GPI-anchored proteins, which are usually glycosylated within their ectodomain. According to UniProt reference database of *Homo sapiens* (date: 2016-06-30), 92% of all transmembrane type I (1125 out of 1228, term: SL-9905) and 83% of all transmembrane type II (347 out of 420, term: SL-9906) proteins are annotated as glycoproteins (term: KW-0325). Upon membrane protein cleavage, a part of the ectodomain is secreted (Figure 2). Glyco-capturing (Figure 3) facilitates specific enrichment of glycoproteins. In the first step, cis-diol groups of N- and/or O-linked carbohydrates are oxidized to aldehydes using periodate. At 1 mM periodate mainly sialic acid residues are oxidized whereas all cis-diol groups can be oxidized with higher concentrations such as 20 mM. The aldehydes can be covalently coupled to a hydrazide resin (Zhang et al., 2003). Alternatively, amino-oxybiotin can be used to biotinylate the oxidized sugars for subsequent pull-down with avidin or streptavidin beads (Zeng et al., 2009). Both, hydrazone and oxime ligations are catalyzed by aniline (Dirksen and Dawson, 2008). After glycoprotein pull-down, proteins are digested with trypsin for MS analysis.

When glycoproteins are covalently coupled to a hydrazide resin, the digestion is performed directly on the beads. In this case, the remaining glycosylated peptides can be released using peptide-N-glycosidase F (PNGaseF) and analyzed as separate fraction to further reduce the sample complexity (Zhang et al., 2003; Stützer et al., 2013). For example, this technique was used to identify substrates of BACE1 and 2 in pancreatic β-cells by quantification of N-glycopeptides of cell supernatants and lysates (Stützer et al., 2013). Glycoprotein labeling is even possible on the cell surface of living cells (Wollscheid et al., 2009; Zeng et al., 2009). Here, glycoproteins are labeled covalently with amino-oxybiotin or biocytin-hydrazide for subsequent enrichment of glycoproteins or glycopeptides using streptavidin beads.

A drawback of glyco-capturing is that secretome analyses usually have to be performed under serum-free conditions because many serum proteins, such as immunoglobulins, are also glycosylated. Thereby, peptides from secreted proteins might be masked by the presence of high abundant peptides from serum protein. However, glyco-capturing might also be used to identify protease substrates *in vivo* using plasma or CSF samples (Table 1).

**Table 1:** **Advantages and disadvantages of different techniques for membrane protease substrate identification**.

	Advantages	Disadvantages
Glycocapture	• Large reduction of sample complexity when analyzing glycopeptides after PNGaseF release. • Possible with *in vivo* material	• Serum-free or serum-depleted medium required
SPECS	• Compatible with protein and serum supplements in the medium • Facilitates secretome and surfaceome analysis. • Many peptides for quantification	• No direct protease cleavage site identification (only semitryptic peptides can be used). • Only applicable for sheddases • Not suitable for *in vivo* analyses
AHA labeling	• Compatible with protein and serum supplements in the medium • Facilitates secretome and surfaceome analysis • Many peptides for quantification	• No direct protease cleavage site identification (only semitryptic peptides can be used) • Only applicable for sheddases • Titration of AHA concentration necessary to prevent toxicity • Not suitable yet for *in vivo* analyses
Surface Biotinylation	• Efficient pull-down of cell surface proteins • *In vivo* analyses are possible	• Secretome analysis difficult • No trypsin cleavage at biotinylated lysines
Murine CSF	• *In vivo* • Many peptides for quantification	• Low sample amount (5–15 μl) • Sampling is difficult (blood or cell contamination) • KO mice or inhibitor treatment of mice is necessary
TAILS	• Direct identification of protease cleavage sites • Also applicable for soluble proteases (e.g., *in vitro* incubation of protein lysate with protease of interest)	• Serum-free or serum-depleted medium required • TAILS is hard to establish (especially C-TAILS) • Few peptides for quantification → Additional whole secretome analysis of labeled peptides potentially required
COFRADIC	• Direct identification of protease cleavage sites • Also applicable for soluble proteases (e.g., *in vitro* incubation of protein lysate with protease of interest)	• Serum-free or serum-depleted medium required • HPLC is required for extensive sample fractionation • Histidine containing peptides are lost in the SCX depletion step • Few peptides for quantification → Additional whole secretome analysis of labeled peptides potentially required
Subtiligase	• Direct identification of protease cleavage sites • Also applicable for soluble proteases (e.g., *in vitro* incubation of protein lysate with protease of interest)	• Serum-free or serum-depleted medium required • Large sample amount required • Few peptides for quantification → Additional whole secretome analysis of labeled peptides potentially required

#### Secretome Protein Enrichment with Click Sugars (SPECS)

SPECS was developed to overcome the difficulty of secretome analysis in the presence of serum or other protein containing culture media additives (Kuhn et al., 2012; Figure 3). For example, primary neurons have to be cultured using additives such as B27 which have a high protein concentration, in particular of albumin. Quantitative proteomics usually covers a dynamic concentration range of 3–4 orders of magnitude. Fetal bovine serum (FBS) has a total protein concentration of 30–45 mg/ml which includes 17–34 mg/ml of albumin (FBS-BBT, Rocky Mountain Biologicals, Inc., Missoula, MT, USA). Thus, conditioned cell culture medium with 10% FBS contains 1.7–3.4 mg/mL whereas the concentration of secreted proteins is three orders of magnitude lower (in the μg/mL range). For example, a concentration of 7.0–7.5 μg/mL was reported for the J774 murine macrophage cell line cultured in 20 mL serum-free medium (Chevallet et al., 2007). Therefore, high concentrations of protein supplements lead to a dramatically decreased quantification of cell-derived secreted proteins.

For SPECS, azido sugars are used for metabolic labeling of glycoproteins in cell culture. ManNAz (N-azidoacetylmannosamine-tetraacylated) is taken up by cells, converted to N-azidoacetyl-sialic acid and mainly incorporated into N-linked glycosylation but also into O-glycosylation (Sletten and Bertozzi, 2011). GlcNAz (N-azidoacetylglucosamine-tetraacylated) and GalNAz (N-azidoacetylgalactosamine-tetraacylated) are primarily used to label O-glycans. After metabolic labeling for usually 24–48 h, shed glycoproteins in cell supernatant and/or surface glycoproteins are modified by copper-free alkyne-azide click chemistry with dibenzocyclooctyne (DBCO)-containing biotinylation reagents. The biotinylated glycoproteins can be efficiently pulled down with avidin- or streptavidin-coupled beads for further analysis. The protocol requires SDS-PAGE fractionation of purified glycoproteins and in-gel digestion because bovine albumin within the conditioned medium cannot be completely removed. However albumin abundance is reduced more than 50-fold and several hundred glycoproteins were identified in the cell supernatant (Kuhn et al., 2012). Alternatively, on-bead tryptic digestion of secreted glycoproteins was reported for low-serum conditions using alkyne beads for covalent coupling (Roper et al., 2013). Here, the secretome of stromal cell lines was directly analyzed under serum-free conditions and compared to glycoprotein enrichment after ManNAz labeling using serum-free and low serum (1%) conditions. Overall, only 75 and 100 proteins were identified using SPECS at serum-free and low serum conditions, respectively. Compared to the whole secretome digestion (193 proteins), a significant enrichment for glycoproteins was reported and 46 additional proteins with lower abundance could be identified.

SPECS has been used to identify substrates of BACE1 (Kuhn et al., 2012) and ADAM10 (Kuhn et al., 2016) in murine primary neuronal cell cultures and of SPPL3 in two different cell lines (Kuhn et al., 2015). SPECS is a technique that is well-suited for substrate identification of sheddases in any cell culture system. Quantification is performed on many peptides in contrast to enrichment of neo N- or C-termini which relies on quantification by one or two peptides per protein. Hence, SPECS offers increased reliability of protein quantification. Yet, as the method does not enrich specifically terminal peptides, cleavage sites cannot be automatically inferred from the MS data (Table 1). However, for some substrates semi-tryptic peptides were identified and allowed determination of the cleavage site.

Importantly, SPECS identifies secreted, cell-derived proteins, regardless of whether they are soluble, secreted proteins or proteolytically derive from membrane proteins. Thus, SPECS can also be used for the identification of secreted proteins as biomarkers. When the research goal is to identify sheddase substrates, the hit list is simply filtered for membrane proteins and thus yields the list of substrate candidates.

A variant of SPECS has also been used to label membrane proteins at the surface of ADAM10-deficient neurons. Compared to wild-type neurons, a number of membrane proteins were found to be enriched, suggesting that they may be ADAM10 substrates. In fact, several of them also showed reduced ectodomain release into the conditioned medium and were validated as ADAM10 substrates (Kuhn et al., 2016). While changes in the secretome are mostly used to identify shedding substrates, these results demonstrate that the enrichment of substrates in the membrane may be an alternative approach.

#### Azidohomoalanine Labeling

Azidohomoalanine (AHA) labeling (Dieterich et al., 2006) is an alternative labeling method of newly synthesized proteins and is similar to the SPECS method. AHA is an azide-containing analog of methionine which is incorporated into proteins via the methionyl-tRNA more slowly than methionine (Kiick et al., 2002). Eichelbaum et al. (2012) established a proteomic method using AHA labeling for secretome analysis in the presence of serum supplements. AHA labeled proteins in the secretome are covalently bound to alkyne resin via Cu(I) catalyzed cycloaddition reaction between azide groups and a terminal alkyne. After stringent washing of the beads, an on-bead digestion is performed. Proteolytic peptides are fractionated followed by LC-MS/MS analysis. This method was used in combination with pulsed stable isotope labeling by amino acids in cell culture (SILAC) to monitor protein synthesis and secretion during macrophage activation (Eichelbaum and Krijgsveld, 2014). This method might also be used with copper-free alkyne-azide click chemistry with DBCO similar to SPECS. While SPECS enriches for N-glycosylated proteins, AHA labeling facilitates capturing of all secreted proteins. However, a drawback is that cellular toxicity has been observed for AHA labeling, which is not the case for azido-sugar labeling. The reason appears to be that AHA, which is not identical to methionine, but is incorporated into the amino acid backbone of proteins, may slightly alter the conformation of numerous cellular proteins leading to cellular toxicity. In contrast, the modified sugars in SPECS are located at the outside of the protein structure and are less likely to affect protein conformation. For the AHA method a careful titration of the AHA concentration can minimize the cellular toxicity for every cell type. The AHA method has not yet been used for protease substrate identification, but may be well suited for determining sheddase substrates. Compared to the other methods for protease substrate identification, AHA labeling and SPECS share similar advantages and disadvantages (Table 1).

#### Surface Biotinylation

An alternative approach for the enrichment of cell surface proteins is their biotinylation using N-hydroxysuccinimide (NHS) chemistry. Proteins are labeled at amino-groups of lysine residues and protein N-termini with NHS-biotin for subsequent pull-down. Even though cell surface biotinylation is frequently used for proteomics, there are no publications for membrane protease substrate identification available. However, this technique was already used to validate MS-based substrate identifications via immunoblotting (Stützer et al., 2013). Unfortunately, this approach is not suitable for secretome analysis because all proteins within the conditioned medium would be labeled, i.e., also serum proteins and not just the cell-derived proteins (Table 1). Additionally, labeled lysine residues are no more accessible for tryptic cleavage which results in long peptides. To overcome this latter issue, peptides can be further digested with other proteases such as GluC to get more peptides with a suitable length for LC-MS analysis.

#### Cerebrospinal Fluid Proteomics

CSF is the only body fluid that is in direct contact with the brain. Therefore, CSF proteomics is the only method that facilitates *in vivo* secretome analysis of the brain. Ideally, KO mice are the system of choice to study membrane proteases, as the proteolytically released substrate ectodomains will be found in the CSF. While milliliters of CSF can be sampled from humans, only 5–15 μl can be collected from mice (Liu and Duff, 2008). This makes proteomic analysis of murine CSF challenging.

Furthermore, sampling of murine CSF is very susceptible to contaminations by cells or blood. The quantification of more than 50% of all CSF proteins is affected even by low levels of blood contamination (Aasebø et al., 2014). Therefore, sampling of murine CSF is the most critical part of the proteomic workflow (Table 1).

An immunodepletion kit from Agilent is available for three most abundant proteins in murine blood which might also work for murine CSF. However, depletion or even fractionation of CSF can lead to sample losses, especially for minute sample amounts. Many CSF proteins bind to albumin and get co-depleted (Holewinski et al., 2013) or might bind unspecifically to the depletion beads or plastic. Additionally, multi-use of immunoaffinity depletion columns require efficient stripping of bound proteins to reduce sample carryover and to maintain the binding capacity (Gundry et al., 2009). Another study performed a proteomic analysis of murine CSF that was immunodepleted with IgY-14 resin which is designed to remove the 14 most abundant human serum proteins (Cunningham et al., 2013). Yet, 100 μl of pooled CSF was used, but overall only 289 proteins were identified. Consequently, direct in-solution digestion without depletion is the method of choice for murine CSF.

Recently, the workflow for murine CSF proteomics was optimized and allowed identification and label-free quantification of BACE1 substrates in mouse brains using individual wild-type and BACE1^−/−^ mice (Dislich et al., 2015). This shows the suitability of this approach to identify protease substrates *in vivo* by proteomics (Table 1). Additionally, it was shown that quantitative CSF proteome analysis of individual mice is possible using only 5 μl CSF resulting in 522 relatively quantified proteins. This was a considerable improvement in comparison to 128 identified proteins from individual mice (Smith et al., 2014) and 103 relatively quantified proteins using pooled murine CSF (Cunningham et al., 2013) in previous studies.

### Methods for Protease Substrate Identification and Cleavage Site Determination

#### Terminal Amine-based Isotope Labeling of Substrates (TAILS)

Protease cleavage creates neo N- and C-termini. If the protease of interest is inhibited or knocked-out, the neo N- and C-terminal peptides are no longer generated. Thus, identification of neo N- and C-terminal peptides allows both substrate and cleavage site identification at the same time. Besides cleavage site determination, the methods for N-termini identification are also suitable for identification of N-termini of whole proteins (Vaca Jacome et al., 2015; Berry et al., 2016). Terminal amine-based isotope labeling of substrates (TAILS) is a method for specific enrichment of the terminal peptides. Two different protocols for enrichment of either protein/peptide N- or C-termini, called N- and C-TAILS are available (Schilling et al., 2010; Kleifeld et al., 2011). The first step of N-TAILS is labeling of α- and ε-amines with methyl groups (dimethyl labeling) or other amino group-reactive isobaric labeling reagents for proteomics, such as iTRAQ or TMT reagents (Figure 4). All free protein/peptide N-termini including the neo N-termini as well as lysine residues are modified. Up to three different conditions can be relatively quantified by using stable isotope dimethyl labeling (Boersema et al., 2009) while up to 10 samples can be relatively quantified with isobaric labeling.

After labeling of amines, samples from the different conditions, i.e., protease inhibition and vehicle control, are mixed. In the next step, the labeled proteins and peptides are digested by trypsin and/or other endoproteases. This leads to peptides derived from the former N-term, the C-term, as well as internal regions called “internal peptides”. The peptides of the former N-termini have no free amino group whereas all other proteolytic peptides have a new, free amino group. Dendritic polyglycerol aldehyde polymers are used to remove all “internal” peptides with free amino groups under mild reductive conditions using cyano-borohydride. In the last step, the remaining N-terminal peptides are analyzed by MS.

C-TAILS is the counterpart of N-TAILS which facilitates the identification of neo C-termini. After dimethyl-labeling, similar to the N-TAILS protocol, carboxyl groups are protected with ethanolamine. A tryptic digestion is used to generate new free N- and C-termini of “internal” peptides. Again, labeling of α-amines is performed. The “internal” peptides are removed by coupling the free carboxyl groups to a polyallylamine polymer. The remaining C-terminal peptides are analyzed by MS.

TAILS is a powerful technique to identify the exact cleavage site of a protease. However, the methods require working at serum-free or low-serum conditions in cell culture for secretome analysis. For example, secreted proteins of different cell lines, which were cultured in serum-free medium, were incubated with recombinant meprin α and β for substrate identification by N-TAILS (Jefferson et al., 2011, 2013). On the other hand, *in vivo* analysis of proteins of cell lysates or membrane fractions is possible (Sabino et al., 2015; Prudova et al., 2016). Yet, quantification is based on only one peptide at the N- or C-terminus of the cleavage site and additionally the N-terminal peptide of the intact protein. To overcome this issue, routinely a whole secretome analysis using the labeled peptides after proteolytic digestion is performed which is used for relative protein quantification (Prudova et al., 2016). Moreover, for C-TAILS it has been difficult to achieve complete labeling of the carboxy-terminal groups, which is a disadvantage for the analysis of type I membrane protein shedding substrates, where the N-terminal ectodomain is released into the conditioned medium and would need to be detected with C-TAILS (Table 1).

#### Combined Fractional Diagonal Chromatography (COFRADIC)

Combined fractional diagonal chromatography (COFRADIC) is an umbrella term for different multistep chromatographic methods that include peptide derivatization, fractionation and isolation of modified peptides. With different types of modifications, it is possible to separate terminal peptides from neo N- and C-termini from other peptides (Van Damme et al., 2010). Usually, two conditions, with and without protease are differentially labeled in cell culture with SILAC using isotopic labeled arginine. Proteins are reduced, alkylated at cysteines and acetylated with NHS-(D3)acetate at α- as well as ε-amines. After tryptic digestion, N-pyroglutamate residues are enzymatically removed by pyroglutamyl aminopeptidases (Abraham and Podell, 1981) because peptides carrying those residues are usually not charged at pH 3.

Strong cation exchange (SCX) cartridges are used at pH 3 to remove the majority of peptides with a free N-terminus, while most peptides with acetylated N-termini have a net-charge of zero because the C-terminal arginine is positively charged but the carboxyl group at the C-term is mostly deprotonated (Staes et al., 2008; Figure 4). C-terminal peptides, which contain no C-terminal arginine are also not positively charged at pH 3 and elute with the flow-through (Staes et al., 2008). Exceptions are histidine containing peptides, because histidine residues are positively charged at a pH of 3. Thus, those peptides are retained by SCX cartridges.

In the next step, hydrogen peroxide can be used to uniformly oxidize all methionines. Now, peptides are fractionated by C18 RP chromatography. The free α-amines of C-terminal and internal peptides in all fractions of both conditions are either differentially labeled with isotopic variants of NHS-butyrate (^12^C_4_, ^13^C_4_) for isolating both (neo) C- and N-terminal peptides. On the other hand, also isobaric tags might be used to label primary amines. Alternatively, free α-amines can be labeled with 2,4,6-trinitrobenzenesulfonic acid (TNBS) which introduces a very hydrophobic trinitrophenyl label, for isolating (neo) N-terminal peptides only (Staes et al., 2011).

In the former, the matching fractions of condition 1 and 2 are combined and a second C18 RP chromatography run is used for isolating terminal peptides for LC-MS based quantification. For TNBS labeling, trinitrophenyl containing internal or C-terminal peptides elute later than in the first C18 RP run which allows efficient separation of the (neo) N-terminal peptides derived by protease cleavage.

Relative quantification of N-terminal peptides is done by SILAC labeling with arginine whereas quantification of C-terminal peptides is based on the butyrate (or alternative) labeling. Different studies have been carried out to identify substrates and cleavage specificities of proteases. For example, substrate specificities of the granzyme tryptases A and K were identified (Plasman et al., 2014). In a more general approach, the secretome of gastric cancer-associated myofibroblasts was analyzed and identified activation of matrix metalloproteinases (Holmberg et al., 2013).

COFRADIC facilitates the identification and quantification of neo N- and C-termini which allows identification of protease substrates and their cleavage sites. However, extensive HPLC fractionation as well as LC-MS analysis of many fractions is very time-consuming. Additionally, histidine containing peptides are lost during the SCX chromatography step (Table 1).

#### Subtiligase Method

The subtiligase protocol enables enrichment of free protein N-termini as well as protease cleavage derived neo N-termini (Figure 4). A peptide ester which contains a biotin, a TEV cleavage site and an Abu-tag (α-aminobutyric acid) is enzymatically coupled to free protein N-termini with subtiligase. The reaction is specific for α- over ε-amines (Braisted et al., 1997). Mostly neo N-termini are modified because 68% of the yeast and 85% of the human proteins are acetylated at the protein N-term (Van Damme et al., 2011). Labeled proteins/peptides are pulled down with avidin or streptavidin conjugated beads. After tryptic digestion and washing, N-terminal peptides are released using TEV protease. The Abu-tag enables the discrimination between labeled N-terminal and background peptides. Quantification can be done label-free or with other label-based methods (Wiita et al., 2014). Like with TAILS and COFRADIC, exact cleavage sites of proteases can be analyzed. A drawback of the method is that typically a high protein amount of 30–300 mg of cell lysate is used according to Wiita et al. (2014; Table 1). The subtiligase method was used for different studies, such as to identify caspase substrate profiles (Agard et al., 2010) and to analyze cell apoptosis (Crawford et al., 2013).

### Summary of Methods

All described methods with the exception of CSF analysis are suitable for any cell culture experiment including primary cells as well as cell lines or bacterial cells. However, cells that require serum or other high protein containing supplements are best analyzed using SPECS or AHA labeling to enrich selectively for cell derived proteins. The other methods are better suited for serum free or low serum conditions.

*In vivo* samples can be analyzed using all methods except the metabolic labeling methods SPECS and AHA labeling which would cause extensive costs for *in vivo* labeling. In the case of body fluids, glyco-capturing has the advantage to enrich for glycosylated proteins which include 89% of type 1 and 2 transmembrane proteins according to UniProt.

TAILS, COFRADIC and the subtiligase method have the advantage to facilitate protein cleavage site determinations. Thus, those methods are well suited for cell-free *in vitro* cleavage assays such as incubation of cell secretomes, potential substrates, peptide libraries, or even whole cell lysates with the protease of interest. Such an approach was reported e.g., for meprin α and β substrate identification by N-TAILS (Jefferson et al., 2013).

## Identification of Membrane Protease Substrates Using Proteomics

In the following paragraphs we will describe the application of several of the proteomic methods described above to the identification of substrates for the Alzheimer-related proteases BACE1 and BACE2, ADAM10, ADAM17 as well as γ-secretase and its distant homolog SPPL3.

### β-Site Amyloid Precursor Protein Cleaving Enzyme (BACE) 1 and 2

The beta secretase BACE1 is known to shed the ectodomain of APP which leads to the release of the sAPPβ fragment. Subsequently, cleavage of the APP C-terminal fragment (CTF) by γ-secretase generates amyloid β peptides which can form plaques in the brain, a pathological hallmark of AD (Selkoe and Hardy, 2016). Therefore, BACE1, which is highly expressed in neurons, is a major drug target to inhibit Aβ generation and thus delaying or preventing the onset of AD. Different pharmaceutical companies have developed BACE1 inhibitors (Vassar, 2014). However, several BACE1 inhibitors have failed in the clinic because of side effects that may not be related to BACE1 inhibition (Barão et al., 2016). The inhibition of BACE1 might also lead to mechanism-based side-effects because it also cleaves other transmembrane proteins. This is further emphasized by the finding that BACE1^−/−^ mice show various phenotypes (Vassar, 2014). Hence, it is essential to identify BACE substrates and to characterize the biological function of the full-length proteins as well as the resulting BACE1 cleavage products.

In recent years, different MS-based proteomic studies were carried out with the goal to identify BACE substrates in an unbiased manner. In 2009, Hemming et al. performed a study with HEK and HeLa cells overexpressing BACE1 and compared the secretome with cells transfected with a control vector (Hemming et al., 2010). Cells were cultured and metabolically labeled in serum-free SILAC medium to enable a MS-based secretome analysis. This study identified 69 putative BACE1 substrates (65 TM type I, 1 TM type II and 3 GPI anchored proteins) that were enriched in the secretome of HEK and/or HeLa cells upon BACE1 overexpression. Different hits were further validated by immunoblotting.

A similar approach was used in a different study (Ivankov et al., 2013). However, even though well-validated BACE1 substrates such as APP, APLP1 and APLP2 were identified, overexpression of BACE1 is known to lead to artificial cleavage of some membrane proteins. One reason is that overexpressed BACE1 can be active in the endoplasmic reticulum (Huse et al., 2002), whereas under endogenous conditions it cleaves in acidic cellular compartments such as trans-Golgi network and endosomes (Vassar et al., 1999). One example is the protein LRP1, which was identified as a BACE1 substrate candidate upon BACE1 overexpression (von Arnim et al., 2005), but did not show any change in cleavage upon inhibition of endogenous BACE1, at least not in primary neurons (Kuhn et al., 2012).

In 2012, two proteomic studies were published which used primary neurons treated with a BACE inhibitor to identify proteins with reduced abundance in the secretome. Thus, these studies were based on endogenous levels of BACE1 and its substrates. The study of Zhou et al. (2012) used primary neurons cultured in Neurobasal medium without protein supplements. N-propionylation was used to differentially label secreted proteins of control and BACE inhibitor treated samples. Finally, 13 putative BACE substrates could be identified that showed reduced abundance in the secretome of inhibitor treated neurons. Additionally, several experiments were carried out to validate L1 and CHL1 as BACE1 and γ-secretase substrates.

For the second study of 2012, SPECS was used for primary neurons treated with a BACE inhibitor or DMSO (Kuhn et al., 2012). This led to the identification of 34 BACE substrate candidates. Seven of them were also validated by immunoblots of BACE inhibitor treated neurons, BACE1^−/−^ neurons and BACE1^−/−^ brain homogenates.

One of the substrates identified in both proteomic studies in 2012 is the cell adhesion protein CHL1. Subsequent studies further validated CHL1 as a BACE1 substrate *in vivo* and demonstrated that BACE1-cleavage of CHL1 is required for correct axon targeting in the olfactory bulb and the hippocampus of mice (Hitt et al., 2012; Barão et al., 2015).

Another proteomic study used the pancreatic β islet cell line Min6 to identify BACE1 and BACE2 substrates (Stützer et al., 2013). This study employed Min6 cells that were overexpressing BACE1 and/or BACE2, cells with single or double KDs of BACE1 and BACE2 as well as control cells. Substrates were identified by glyco-capturing. For validation of hits from the first screen, the results of seven proteins were further validated by immunoblotting. The same study also used primary islets from BACE1^−/−^, BACE2^−/−^ and BACE double KO (DKO) mice as well as BACE inhibitor treated islets for validation. Relative protein quantification of 56 candidates was performed using targeted proteomics (selected reaction monitoring). Finally, 40 candidates showed an accumulation in cell lysates and/or reduced abundance in cell supernatants (≥1.25-fold) in at least one of the BACE KO or inhibition conditions. The proteins SEZ6L, SEZ6L2 and TMEM27 were further validated as BACE2 substrates in murine primary islet cells (WT vs. BACE1^−/−^, BACE2^−/−^ and BACE DKO) by immunoblotting.

Finally, a label-free quantitative proteomic analysis of CSF from BACE1^−/−^ was performed to identify BACE1 substrates *in vivo* (Dislich et al., 2015). In this study, 10 BACE1 substrates or substrate candidates showed to have a significantly lower abundance in BACE1^−/−^ CSF (APP, APLP1, APLP2, CHL1, CNTN2, NCAM1, PLXDC2, PAM, PTPRN2, SEZ6L2) indicating that CSF proteomics is able to identify and validate BACE1 substrates *in vivo*. Furthermore, APLP1 and 2 were validated by immunoblotting and PTPRN2, PLXDC2 as well as ENPP5 were confirmed by *in vitro* assays as BACE1 substrates.

Taken together, the long list of BACE1 substrates demonstrates a central role for BACE1 in basic neurobiology. Whether the substrates and their functions have an impact on the suitability of BACE1 as a drug target in AD, remains to be carefully monitored in future studies.

### ADAM10

ADAM10 acts as the constitutively active APP α-secretase and is a drug target for AD (Jorissen et al., 2010; Kuhn et al., 2010; Saftig and Lichtenthaler, 2015). ADAM10 is a ubiquitously expressed metalloprotease of the adamalysin family that regulates through shedding the function of several transmembrane proteins, thereby playing a crucial role in cell-signaling and development. The early embryonic lethality of ADAM10-deficient mice has been associated with loss of Notch signaling, that emerged to be a major ADAM10 substrate (Hartmann et al., 2002). Moreover, mice with a conditional knock-out of ADAM10 in neurons, show postnatal lethality at about 3 weeks and display numerous phenotypes in the brain, including impaired synaptic function and disorganized laminar architecture of the neocortex. However, the underlying substrates were largely unknown. A recent study used SPECS and identified around 90 substrate candidates for ADAM10 in primary murine neurons (Kuhn et al., 2016). Several of them were validated by immunoblots. One of the substrates is the cell adhesion protein NrCAM for which it was demonstrated that its loss of cleavage in ADAM10-deficient mice correlates with deficits in axon targeting in the olfactory bulb in mouse brains (Kuhn et al., 2016). More of the newly identified ADAM10 substrates are likely to be assigned in the future to the numerous phenotypes in ADAM10-deficient mice and will enhance our understanding of the broad neurobiological functions of this protease.

### ADAM17

The metalloprotease ADAM17 is a homolog of ADAM10. When activated, it can act as an additional α-secretase and may reduce Aβ levels (Caccamo et al., 2006). ADAM17 is also known as TNF-α converting enzyme (TACE) and was the first sheddase to be identified, as the enzyme responsible for releasing the soluble ectodomain of TNF (Moss et al., 1997). ADAM17 plays a crucial role in cell-cell communication, being able to release not only TNF, but also several other transmembrane proteins, including cytokines, adhesion molecules, receptors and growth factors. ADAM17-deficient mice display several abnormalities at birth, including open eyes and skin defects, that phenocopy mice lacking EGF receptor or a number of its ligands, which are known substrates of ADAM17 (Blobel, 2005). Most ADAM17 substrates have been identified through candidate approaches (Qian et al., 2016). Proteomics has not been extensively used to uncover ADAM17 substrates. However, a few secretome analyses have been performed which searched for transmembrane proteins undergoing shedding in response to specific stimuli, such as lipopolysaccharide (LPS) and 12-O-tetradecanoylphorbol 13-acetate/Phorbol 12-myristate 13-acetate (TPA/PMA), which are known to also activate ADAM17. One study identified a number of transmembrane proteins, such as CSF1R and Sema4D, that are shed by metalloproteinases in response to LPS or TPA in macrophage-like cells (Shirakabe et al., 2014). In order to investigate proteomic changes induced by LPS in macrophages, one study used a method similar to SPECS (Eichelbaum and Krijgsveld, 2014), whereas another group performed secretome analysis from a small number of cells cultured without serum (Meissner and Mann, 2014). Together with a list of known substrates of ADAM17, these studies identified a number of proteins that can also potentially be cleaved by ADAM17. However, these proteins were not validated as ADAM17 substrates so far. Another study specifically investigated changes in the secretome of ADAM17^−/−^ mouse embryonic fibroblasts (MEFs) by using SILAC or label-free based approaches (Kawahara et al., 2014). Label free secretome analysis identified 179 proteins, which were significantly down-regulated in ADAM17-deficient MEF cell supernatants. Transmembrane proteins, including TNFR2 and syndecan-4, were strongly reduced in the secretome of ADAM17^−/−^ MEFs, suggesting that they are ADAM17 substrates. Furthermore, a proteomic study of ADAM17-deficient epidermis was performed which showed pronounced changes in a number of proteins involved in barrier formation, including transglutaminases, involucrin, filaggrin and filaggrin-2 (Tholen et al., 2016).

Functions of ADAM17 in the brain have been little explored so far and no proteomic study has as yet been done to address this issue specifically. Yet, the role of ADAM17 in inflammation suggests that ADAM17 is also involved in various neuroinflammatory conditions.

### γ-Secretase

γ-secretase has been a major drug target in AD in the past. It is a protease complex that cleaves transmembrane type 1 proteins within or close to their transmembrane domain. While γ-secretase only directly sheds the ectodomain of a single, naturally short substrate (Laurent et al., 2015), it typically requires shedding of its substrates in order to cleave them within the transmembrane domain. In 2008, a proteomic study was performed to identify γ-secretase substrates in HeLa cells (Hemming et al., 2008). Therefore, cells were differentially labeled with the SILAC method and treated with the γ-secretase inhibitor DAPT or DMSO as a control. Since γ-secretase cleavage usually requires previous shedding by other proteases (Struhl and Adachi, 2000), such as BACE1 or ADAM10, substrates are commonly identified by an accumulation of the CTF upon γ-secretase inhibition. Hence, SDS-PAGE of membrane fractions was applied for proteomic γ-secretase substrate profiling to separate CTFs from full-length proteins (Hemming et al., 2008). The gels were cut into 10 slices and in-gel digestion was performed with trypsin. Relative quantification between DAPT and DMSO was done separately for each fraction. CTFs with a DAPT/DMSO intensity ratio larger than 1.86 were considered as enriched. Overall, CTFs of 13 proteins, among them APP and APLP2 showed enrichment for DAPT treatment. Very likely, this approach missed to identify more γ-secretase substrates as CTFs of proteins with a short cytoplasmic domain are hard to quantify. Additionally, low molecular weight peptides and proteins offer just few tryptic peptides and are often lost during washing steps of the in-gel digestion protocol (Klein et al., 2007; Müller et al., 2010).

### Signal Peptide Peptidase-Like 3 (SPPL3)

The signal peptide peptidase (SPP) family has five members, SPP, SPPL2A, 2B, 2C and 3. They are distant homologs of γ-secretase and belong together with γ-secretase to the intramembrane-cleaving aspartic proteases. The SPP family cleaves type II transmembrane proteins within or close to their transmembrane domain (Voss et al., 2013). Similar to most substrates of γ-secretase, ectodomain shedding by another protease is required to enable cleavage by SPP, SPPL2A and B (Voss et al., 2013), whereas it has not been investigated so far if SPPL2C is proteolytically active and which biological role it has. An exception to the other family members is SPPL3, which does not require prior shedding of its substrates by another protease (Krawitz et al., 2005; Voss et al., 2013). Therefore, type II transmembrane proteins can be directly shed by SPPL3, which is mostly localized in the Golgi apparatus (Krawitz et al., 2005; Voss et al., 2013).

A global secretome analysis of HEK cells overexpressing SPPL3 as well as MEF SPPL3^−/−^ cells were used for unbiased substrate identification (Kuhn et al., 2015). For this purpose, the SPECS method was applied to enrich and quantify secreted glycoproteins. The majority of identified SPPL3 substrates are involved in modifying N- or O-glycosylation. Hence, SPPL3 has a fundamental role in regulating different protein glycosylation pathways. Whether and how this role impacts the brain, needs to be studied in the future.

## Conclusion and Outlook

The first substrates of sheddases and intramembrane proteases were largely identified by candidate approaches, partially driven by the phenotypes of the corresponding protease knock-out mice. One example is the loss-of-Notch-function phenotype which allowed to identify Notch as a substrate for ADAM10 and γ-secretase (De Strooper et al., 1999; Struhl and Greenwald, 1999; Hartmann et al., 2002). Another example is the hypomyelination of BACE1-deficient mice which led to the identification of neuregulin-1 as a BACE1 substrate (Hu et al., 2006; Willem et al., 2006).

In the last 10 years the focus has shifted to the use of proteomics as an unbiased method for the systematic substrate identification of sheddases— on which we focus in this review, but also of other proteases, including metalloproteases in inflammation and caspases in apoptosis. Generally, the field of proteomics dealing with proteases, protease inhibitors and protein degradation is referred to as degradomics (López-Otin and Overall, 2002). Given the advance in mass spectrometric instrumentation and the development of powerful degradomic methods as described here, we are likely to see many more systematic substrate identification studies being published over the next years. This will include many of the over 40 sheddases and intramembrane proteases, where substrates and functions are little understood to date. The degradomics methods are likely to be further improved to allow analysis of lower sample amounts, and analyses will be increasingly done with *in vivo* material, such as a tissue samples and body fluids.

Several of the sheddases and intramembrane proteases—such as BACE1, ADAM10, ADAM17 and γ-secretase—are major drug targets for neurodegeneration or inflammatory diseases. Other proteases of these families will likely turn out to be drug targets for additional diseases. Thus, degradomic studies of these exciting protease families will not only allow us to understand their basic functions in the brain and other tissues, but will also enable us to better evaluate their therapeutic potential and to predict possible side effects of drugs modulating the protease activity. Additionally, the cleaved ectodomains of the protease substrates in body fluids, such as CSF, hold the potential to be used as companion diagnostics for monitoring whether and how patients respond to protease inhibitors. Thus, the new proteomic methods have paved the way for even faster discovery in basic and applied neuroscience and other research fields.

## Author Contributions

SAM, SDS and SFL co-wrote the review.

## Conflict of Interest Statement

The authors declare that the research was conducted in the absence of any commercial or financial relationships that could be construed as a potential conflict of interest.
